# Design of Artificial Enzymes Bearing Several Active Centers: New Trends, Opportunities and Problems

**DOI:** 10.3390/ijms23105304

**Published:** 2022-05-10

**Authors:** Diego Carballares, Roberto Morellon-Sterling, Roberto Fernandez-Lafuente

**Affiliations:** 1 Departamento de Biocatálisis, ICP-CSIC, Campus UAM-CSIC, 28049 Madrid, Spain; diego.carballares@csic.es (D.C.); r.m.sterling@csic.es (R.M.-S.); 2 Student of Departamento de Biología Molecular, Universidad Autónoma de Madrid, C/Darwin 2, Campus UAM-CSIC, Cantoblanco, 28049 Madrid, Spain; 3 Center of Excellence in Bionanoscience Research, External Scientific Advisory Academic, King Abdulaziz University, Jeddah 21589, Saudi Arabia

**Keywords:** promiscuous enzymes, fused enzymes, artificial metal-enzyme composites, plurizymes

## Abstract

Harnessing enzymes which possess several catalytic activities is a topic where intense research has been carried out, mainly coupled with the development of cascade reactions. This review tries to cover the different possibilities to reach this goal: enzymes with promiscuous activities, fusion enzymes, enzymes + metal catalysts (including metal nanoparticles or site-directed attached organometallic catalyst), enzymes bearing non-canonical amino acids + metal catalysts, design of enzymes bearing a second biological but artificial active center (plurizymes) by coupling enzyme modelling and directed mutagenesis and plurizymes that have been site directed modified in both or in just one active center with an irreversible inhibitor attached to an organometallic catalyst. Some examples of cascade reactions catalyzed by the enzymes bearing several catalytic activities are also described. Finally, some foreseen problems of the use of these multi-activity enzymes are described (mainly related to the balance of the catalytic activities, necessary in many instances, or the different operational stabilities of the different catalytic activities). The design of new multi-activity enzymes (e.g., plurizymes or modified plurizymes) seems to be a topic with unarguable interest, as this may link biological and non-biological activities to establish new combo-catalysis routes.

## 1. Introduction

Humankind demands that chemical production should be as sustainable and clean as possible [1,2,3,4,5], and catalysis is a necessary tool to reach this goal [6]. Biocatalysis should have a prominent role in this target [7,8,9,10]. Enzymes are able to catalyze the most complex processes under very mild conditions in a very atom-efficient way [11,12,13,14,15]. This is due to their high activity under atmospheric pressure and room temperature, high specificity (that permits the modification of a single compound in a mixture of very similar compounds) and selectivity (that permits the production of a single product among several possible ones). Nature provides a huge amount of enzymes bearing different catalytic features, and the current development of metagenomics tools has enlarged the range of available enzymes reaching even those produced from non-cultivable or no longer existing organisms [16,17,18,19,20,21]. Enzymes may be further improved to enhance their performance by genetic tools, such as directed evolution [22,23,24,25,26] or site-directed mutagenesis (based in dynamic simulations, enzyme modelling, etc.) [27,28]. Finally, enzyme chemical modification [29,30,31,32] or immobilization [33,34,35,36,37,38] may be the last opportunity of making enzyme features fit specific industrial requirements.

The design of artificial metalloenzymes is a quite mature discipline nowadays [39,40,41,42,43,44,45,46,47]. These artificial enzymes have special relevance when there are no natural enzymes available to catalyze the target reaction. It enables the preparation of protein-based biocatalysts where the metallic component is the catalytically active phase, whereas the enzyme environment may tune its features (providing higher catalytic efficiency, specificity or selectivity) [48,49,50,51].

One step further in the complexity of biocatalytic processes is the design of multi-enzyme processes, which may include cascade reactions [52,53,54,55,56] or the modification of several components of a complex substrate (e.g., hydrolysis of galactose and casein in milk or multiple glycerides contained in oils [57]). These cascade processes include the step by step or simultaneous use of several enzymes. The simultaneous use of several enzymes is preferred, as this reproduces the biological metabolic chains where the enzymes work in vivo, and may be used to shift equilibrium and to reduce inhibitions or inactivations [52,53,54,55,56]. Moreover, this permits the development of one step/one reactor processes, with a corresponding savings in time and resources. However, this may raise some additional problems: the reaction conditions must fit the operational window for all involved enzymes; if some deleterious product is released by one of the enzymes (e.g., hydrogen peroxide), all enzymes will be exposed to it, etc. [58].

Thanks to the huge developments in the diversity of areas involved in the design of enzymes, one of the current trends in performing cascade reactions is the design of artificial enzymes bearing two active centers. This strategy has a distinct advantage. A single peptide chain, produced in a single fermentation, may present the activities required to catalyze the whole reaction chain. This way, just one biocatalyst needs to be produced, purified and immobilized, with the corresponding cost efficiency. The conceptual advances and advantages of these enzymes bearing multiple active centers are obvious, and the potential of some of these strategies (some of them really novel) still need to be properly developed to be fully exploited. However, this can also pose some difficulties for their implementation, which needs to be at least considered.

This review paper intends to quickly summarize the efforts performed to produce these artificial enzymes bearing several active centers, putting more emphasis on the discussion of their possibilities and some practical problems that can be envisioned and should be considered in future developments.

## 2. Enzymes Bearing Several Active Centers

In this section, we will list different examples where enzymes bearing two active centers are created.

### 2.1. Natural Enzymes

**Figure 1:**
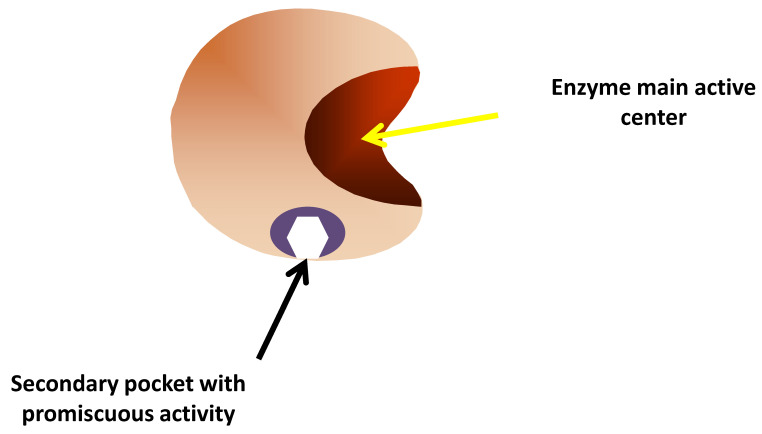
Enzymes having promiscuous activities not related to the main active center.

We have been unable to find reports describing natural enzymes bearing two independently active centers except when some natural fusion enzymes are formed, but these will be treated in the next section. An exception to this fact is the enzymes exhibiting activity promiscuity. Substrate promiscuity is related to enzyme specificity, and it is not a surprise that an enzyme can recognize many different substrates, since many enzymes have low specificity (an outstanding example are lipases, which are able to recognize many different substrates) or can catalyze diverse-related reactions using the same catalytic center that is used for the physiological reaction, as happens in ester hydrolysis, esterification, transesterification and so on [59,60,61]. These are enzymes with a broad specificity and are able to catalyze very diverse reactions. However, here we refer to a promiscuous activity, that may be defined as the capacity of the enzyme to catalyze a reaction far from the physiological activity of the enzyme, that is, with different chemo-selectivity. An example may be a hydrolase able to produce carbon–carbon bonds or exhibiting an oxidative activity. This is the real enzyme promiscuity, and it is postulated that it has been somehow related to the evolution of organisms, as first step before gene diversification to obtain new enzyme activities [62,63,64]. These promiscuous activities may be of interest when there are no alternative natural enzymes able to perform the reaction [65,66,67,68,69,70,71,72,73]. In many examples, it has been shown that this secondary catalytic activity requires a folded enzyme structure (that is, it is not a consequence of the catalytic activity of individual amino acids) but that did not involve the active center of the enzyme (as the activity remains after blocking the active center of the enzyme with irreversible inhibitors) [74,75]. This can suggest that the active center responsible for this activity may be placed in a different position, perhaps in a different pocket of the enzyme surface (Figure 1). As the standard enzyme activity, this promiscuous activity may be modulated by enzyme immobilization [74]. However, the catalytic activity values are usually moderate, and obviously, the main active center and this promiscuous activity may be utilized to catalyze cascade reactions only randomly [76]. Nevertheless, we wanted to mention this possibility as an example of natural enzymes bearing two active centers.

### 2.2. Fusion Enzymes

The building of enzymes bearing a domain has been a traditional strategy to facilitate enzyme purification, producing chimeric enzymes using very simple and small tags, such as poly-His (using immobilize metal chelate columns) [77,78] or poly-Lys tags (to purify the enzyme using anionic exchanges), taking advantage of the usual low isoelectric point of enzymes [79] to large domains that are designed to introduce affinity moieties, such as cellulose binding domain or choline binding domain [80,81]. These affinity domains may also be used as a single step immobilization purification of enzymes [82,83,84,85,86,87,88,89,90,91,92].

However, nature has shown us that this may be also used to produce enzymes bearing several catalytic domains by fusing the domains of different enzymes. One example of this is the covalent fusion of soluble P450 and cytochrome P450 reductase enzymes from *Bacillus megaterium* to produce the flavocytochrome P450_BM3_ system, and to achieve a highly efficient electron transport system for oxygenation of fatty acids and related molecules [93]. This gene fusion seems to be a natural evolutionary event due to fusion of adjacent genes [94,95].

**Figure 2:**
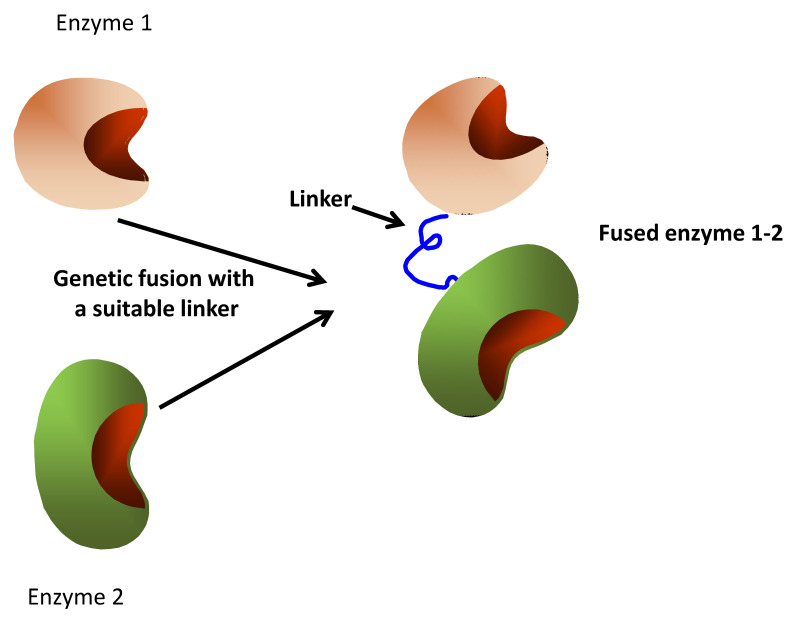
Design of artificially fused enzymes.

Considering the advantages of having a single molecule bearing two desired catalytic activities, researchers have tried to imitate nature and produce fusion enzymes for a long time (Figure 2). These fusion enzymes have been used in biomedicine [96], in vivo to produce target metabolites via fermentation [97,98,99,100,101,102] or as isolated bifunctional enzymes to catalyze cascade reactions [103,104,105,106,107,108,109,110,111,112,113]. The selection of appropriate linkers between the involved enzymes is a critical point in the design of these fusion proteins, to permit a proper folding of both fused enzymes [114]. In some instances, the researchers fused more than two enzymes [115].

These fusion proteins can be produced genetically, fusing coding open reading frames, or the connection of the proteins may be performed in a posttranslational process. This interesting topic has been the subject of many interesting reviews [116,117,118], therefore, we are not going to extend further in this matter.

### 2.3. Modification of Enzymes with Non-Biological Catalysts

The combination of enzyme and metal catalysis is highly interesting and can open new synthetic possibilities. Unfortunately, in many instances the combined use of both catalysts lead to mutual inactivation, making some compartmentalization necessary to prevent this negative effect [119]. However, another alternative is to prepare enzymes bearing both catalytic activities, via the modification of the enzymes with a compound bearing the desired catalytic activity. This has been recently reviewed [120]. Here, we will just give some examples to show the potential.

For example, glucose oxidase was coated with hemin (a peroxidase-mimetic catalytic polymer) via a flexible polymeric scaffold through coordination to their imidazole groups. This spatial distribution allows the enzyme to catalyze its reaction first and then hemin catalyst acts. This was used to build nanoreactors able to degrade organic aromatic compounds using glucose as the only fuel [121].

**Figure 3:**
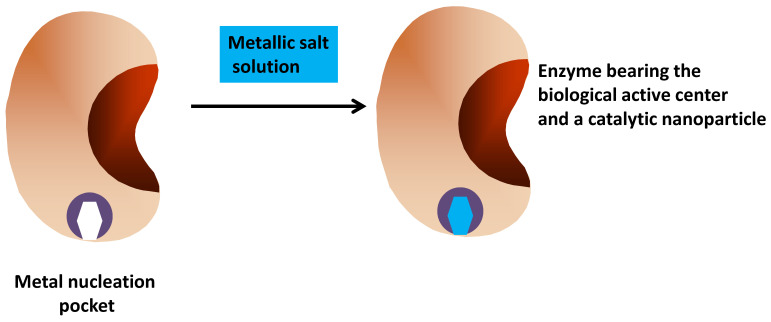
Preparation of an enzyme bearing its biological center and a nanoparticle.

Another strategy consists of using the enzyme as a scaffold to get a metal nanoparticle attached to the enzyme (Figure 3). In fact, the use of different organisms to produce transition metal nanoparticles in a greener way has been proposed by some authors, in vivo or in vitro [122,123,124,125,126,127,128,129,130,131]. The production of nanoparticles employing enzymes as inductors of the metal nanoparticle formation in aqueous media is a well-known possibility [124,132,133]. Thus, it is possible to build enzymes bearing metal nanoparticles to get multifunctional hybrid-enzymes. One example of this may be the use of an alcohol oxidase to produce an in situ nanoparticle, used as an amperometric alcohol biosensor [134]. There are many other interesting examples of this kind of hybrid biocatalyst [135,136,137,138].

**Figure 4:**
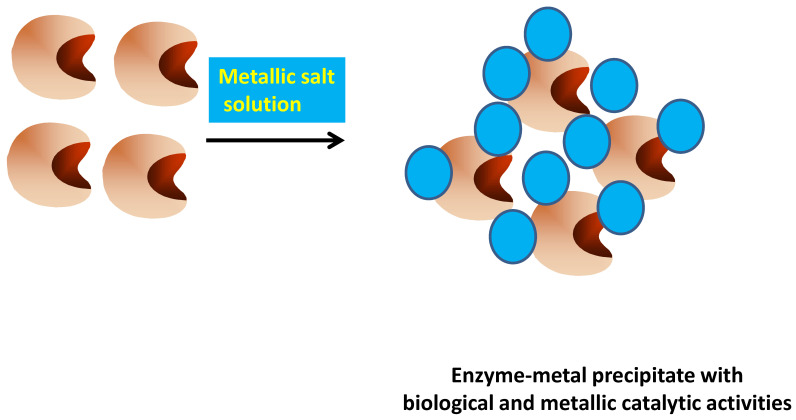
Preparation of metal-enzyme aggregates.

In some instances, this strategy may also solve the problem of the biocatalyst solubility. For example, lipase B from *Candida antarctica* was mixed with different metal salts (Pd(OAc)_2_, Na_2_PdCl_4_, AgNO_3_ and HAuCl_4_), to produce a solid precipitate that could be used as an ex novo heterogeneous biocatalyst [139] (Figure 4).

**Figure 5:**
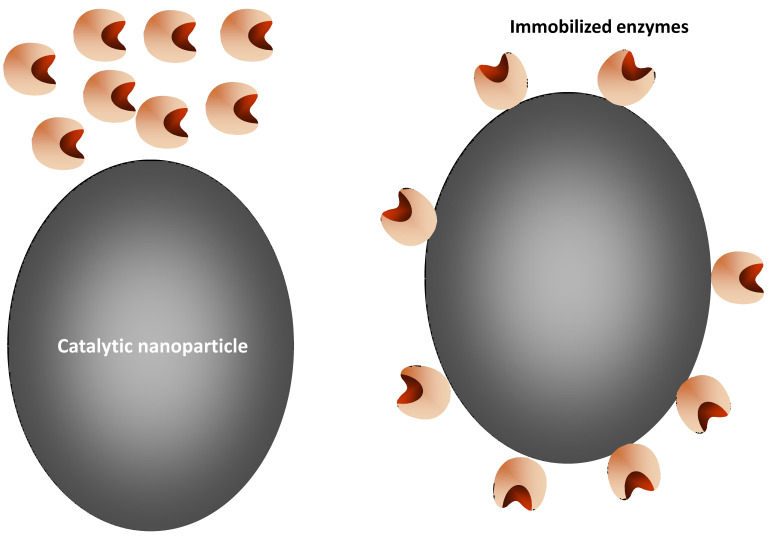
Preparation of enzymes immobilized in metal catalyst nanoparticle.

The enzyme activity recovery depended on the metal. This biocatalyst was utilized in the cascade reaction (enzymatic hydrolysis plus metal catalyzed reduction) for the transformation of *p*-nitrophenyl butyrate to *p*-aminophenol [139]. One obvious alternative to this strategy is to utilize the enzyme immobilized in a nanoparticle containing the metal [140] (Figure 5). While the first strategy utilizes a green method to produce the aggregate in one step, the second permits better control of the enzyme-support interactions, which, as discussed in the introduction section, may permit a better final enzyme stability/activity. Another example of the preparation of a metal/enzyme precipitate is the production of an oxidase precipitate hosting small spherical palladium nanoparticles that present catalytic competence for both the biocyclization as well as the C–C bond-forming cross coupling [141].

**Figure 6:**
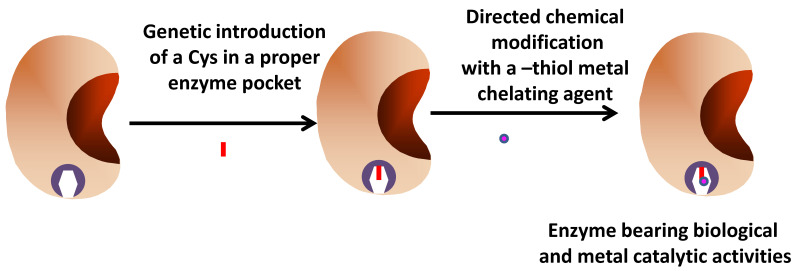
Preparation of enzyme site-directed modified with a metal catalyst.

These strategies may present some problems, because the metal can still be dissolved under certain conditions affecting the enzyme activity and also decreasing the metal activity. The insertion of an organometallic catalyst onto a specific point of the enzyme is a more elegant way to reach the desired goal (Figure 6). In one interesting example, the lipase from *Geobacillus thermocatenulatus* was utilized. This is a peculiar lipase presenting a double lid [142]. They optimized the solid-phase modification of the active center of the enzyme [143], and later on, this protocol was extrapolated to the modification of the position 196 (where a Cys had been genetically introduced), located in the external face of the main lid, with a thiol reactive-organometallic complex. The position where the Cys was introduced was justified by the authors because the introduction on this position of a peptide had previously permitted a strong modulation of the enzyme selectivity, revealing their importance [144]. Very interestingly, the authors showed the great impact of the immobilization strategy on the expressed activity of the introduced organometallic catalyst [143]. This was explained by the necessity of having the open form of the lipase to leave accessible the new catalytic groups. The new immobilized hybrid enzyme was utilized in a cascade reaction to produce aminoarene from a nitroarene ester [143].

Another strategy to create artificial enzymes is the introduction of noncanonical amino acids with catalytic activity in proteins to give some catalytic activities via genetic modification [145,146,147]. When combining this and the previously described tools, in a further effort, a protein may be transformed in an enzyme bearing two artificial active centers. The researchers introduced (on the lactococcal multidrug resistance regulator) a genetically encoded unnatural *p*-aminophenylalanine residue. This group is able to activate an enol via iminium ion formation. They also introduced a supramolecularly bound Lewis acidic Cu(ii) complex (which activates the Michael donor by enolization and supplies it to one preferred prochiral face of the activated enal) [148]. That way the final hybrid biocatalyst was able to act synergistically to achieve high activity and enantioselectivity (up to >99% e.e.) in a catalyzed Michael addition reaction. Using a similar double modification, a biocatalyst able to catalyze the tandem Michael addition/enantioselective protonation highly enantioselective reaction was also prepared [148].

### 2.4. Design of Enzymes Bearing an Ex Novo Biological Active Center (Plurizymes)

**Figure 7:**
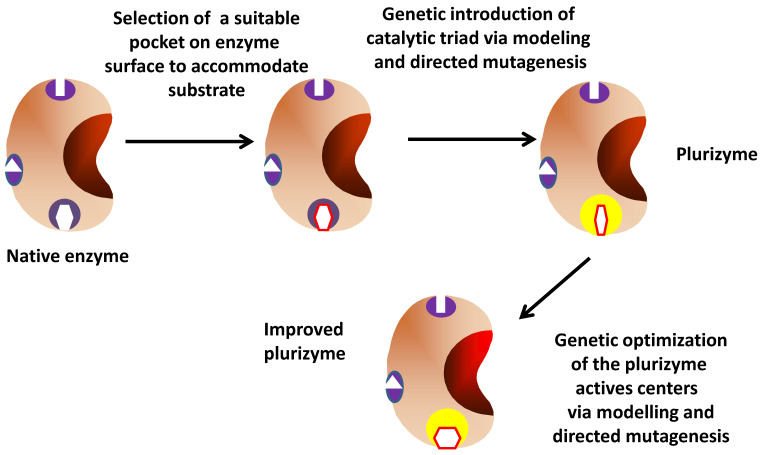
Design and optimization of plurizymes.

The combined use of enzyme modelling, dynamic simulation and genetic tools have enabled the design of the so-called plurizymes by the research group directed by Prof. Ferrer [149]. This novel strategy consists of the search (using enzyme modeling and dynamic simulation) on the surface of an enzyme for pockets that can be suitable to create a new, ex novo, human-designed biological active center (via a minimum of site directed mutations). This ex novo artificial active center will add its catalytic activities to that of the initial active center. That way, an enzyme bearing two biological (one natural and one artificial ones) will be created [149] (Figure 7). The first approach was the introduction of a second serine hydrolase active center (including the whole catalytic triad) in a serine ester hydrolase obtained by metagenomics approaches [150] and previously identified as the ester hydrolase, with the broadest specificity among a total of 147 esterases assayed [151]. This enzyme was studied to identify likely pockets where the new catalytic triad may be accommodated and the substrate may be adsorbed (using tripropionin as a model substrate), and employing Protein Energy Landscape Exploration (PELE) software (because it enables mapping ligand diffusion and binding) [151,152,153,154,155,156]. A pocket on the enzyme surface containing a Ser (residue 211) was identified, and Asp and His residues were first computationally and later experimentally added (Glu25Asp and Leu214His) to generate a catalytic triad. Special care was taken in the distances between the residues and substrate accommodation [149]. The near Gly207, Tyr208 and Phe209 groups generate a likely oxyanion hole.

The natural catalytic Ser of the native enzyme was then mutated (Ser161Ala) to eliminate the native enzyme activity and ensure the functionality of the new active center. The authors found activity of the new active center versus 24 substrates, obviously with different specificities and activities when compared to the native active center. This catalytic activity was dependent on the two mutations introduced to generate the catalytic triad as well as of the natural Ser211. Then, the authors generated an enzyme bearing the two intact catalytic centers. The specific activity of the double active center enzyme for all accepted esters was lower than that observed for the native enzyme, but higher than that for the enzyme bearing only the artificial active center [149]. The enzyme specificity was not enlarged, as the scaffold enzyme was already one esterase bearing a very broad specificity. However, the enzyme bearing the two active centers gave an altered substrate/activity (giving a sigmoidal curve) and pH/activity (giving a narrower peak) curves. This is the only example of a design of plurizymes in the literature to date, and it may be considered a proof-of-concept using relatively simple active centers to facilitate the success. However, it is expected that many other examples may follow this model, perhaps plurizymes, which not only show two different active centers, but also with two different catalytic activities.

In a further step, the model plurizyme features were improved by site-directed mutagenesis [157]. The objective was to achieve better spatial configuration of the active center, and they were able to produce an improved plurizyme with increased catalytic activity (even by 74-folds), an increased enantiospecificity (by over 1000-fold) and an increased temperature of 20 °C, at which point the enzyme retained more than 80% of its optimal activity. It also expanded the substrate scope, as the resulting plurizyme presented activity versus some substrates that were not recognized by the original esterase [157].

### 2.5. Design Plurizymes That Are Site-Directed Modified with Organometallic Catalysts

**Figure 8:**
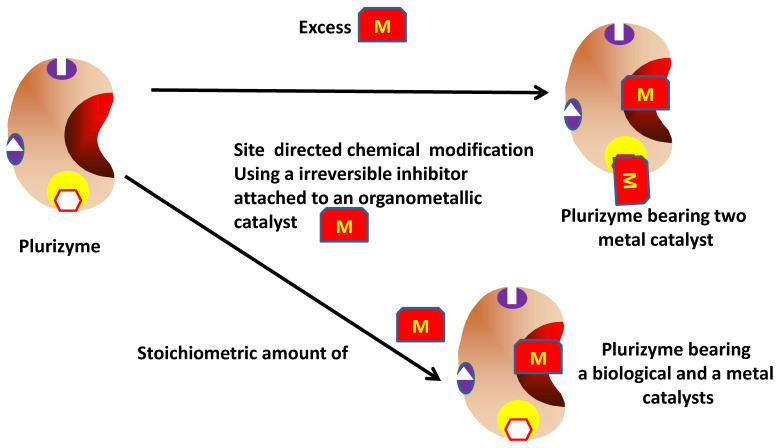
Design and modified plurizymes; single or multiple catalytic activities.

The modified plurizymes described in the previous section were also the first (and only at present) examples of a new strategy to obtain plurizymes bearing a biological and a metal-organic catalytic center [157] (Figure 8). The researchers designed an irreversible serine hydrolase inhibitor attached to an organometallic catalytic complex. To reach this goal, 3′-hydroxy-2,2′-bipyridin-3-yl methyl hexylphosphonate, coupled to a transition-metal-chelating moiety (the bipyridine ligand) was synthesized. The use of an excess of the inhibitor permitted blocking of both native (Ser161) and artificial (Ser211) catalytic groups. The double-blocked enzyme, after incubation with Cu(NO_3_)_2,_ exhibited a synergy in the oxidation of catechol, as the double-modified enzymes were more active than both individually modified enzymes. The authors postulated that the proximity of the two organometallics facilitated the intramolecular electron transfer. In this way, one advantage of using plurizymes was found [157].

However, this composite still has only one catalytic activity bearing two active centers. To have a plurizyme bearing two different activities, the researchers utilized a different enzyme specificity for the different substrates, and therefore, for the inhibitor. They found that the native active center of the plurizyme presented a much higher affinity for the inhibitor than the artificial one. In that way, using almost a stoichiometric amount of inhibitor, they were able to selectively inhibit the native active site while leaving the artificially introduced one unaltered. This new mono-modified plurizyme presented two different catalytic activities and could be utilized as single catalyst of different one pot cascade reactions. For example, it was used to transform 1-naphthyl acetate into 1,4-naphthoquinone (with a conversion near to 100%) and vinyl crotonate and benzene into 3-phenylbutyric acid (with a conversion next to 85% and an enantiomeric excess of 99.9%) [157].

The versatility of this strategy to introduce different active centers, benefitting from the environment generated by the active enter pocket, may open unlimited opportunities to prepare catalysts able to catalyze the most complex processes under the milder and most selective conditions. We foresee a great development in the area of plurizymes and modified plurizymes in the upcoming future.

## 3. Practical Problems of Enzymes Bearing Several Active Centers

The developments to generate artificial enzymes bearing several active centers that have been discussed above are academically very relevant and may open the door to new and unexpected advances in the design of bioprocesses that some years ago seemed only to be a dream. Moreover, some developments are so recent that the full impact that they may have are still unknown, similar to the modified plurizymes. However, we can foresee some problems for the application of these artificial enzymes bearing several active centers.

For example, one critical step in the optimization of multi-enzymatic processes is the optimization of the ratio between the activities of the involved catalytic entities [52,53,54,55,56]. The problem has more or less importance depending on the strategy utilized to have several active centers. Using fusion multi-enzyme composites, it is theoretically possible to add more or fewer enzyme-units to the fusion target enzyme to have the desired activity ratio, or, more simply, to add the required amount of the individual enzyme, which is less active. However, if using the alternatives involving just one enzyme structure where a new active center is created, the optimization may be more difficult. If the activity that needs to be used in excess is that belonging to the native active center of the enzyme, it may be possible to use a mixture of the natural enzyme and the artificial one to get the desired native/artificial activities ratio (assuming that the properties of the natural and the modified enzymes are similar, that may be untrue in many instances). If the activity that needs to be reinforced is that from the artificial active center, to get compensated activities ratios may require using some multi-activity enzymes having the native enzymatic catalytic center inactivated and only bearing the artificial active center. If the artificial activity is less active and it is the second in the chain, it may produce the accumulation of its substrate. If this substrate is unstable (e.g., tends to isomerize or racemize, or it is easily oxidable), the result of the process will not be the desired one, as a percentage of the intermediate product will be destroyed [158,159,160,161,162,163,164,165].

Another likely problem may rise if the stability of one of the activities is much lower than the stability of the other components of the chain. This way, this weak component will mark the stability of the whole composite, causing the same problem that arises when using coimmobilized native enzymes [36,38]. Using coimmobilized enzymes, some solutions have been recently proposed for these dissimilar stabilities of the different enzyme components, designing strategies that permit the release of the least enzyme component after its inactivation and the immobilization of a fresh enzyme batch [58,166,167,168,169,170,171,172,173,174]. This solution is not valid using enzymes bearing multiple activities; the only solutions will be as described above to get a balanced ratio between the involved activities.

One exception may be (as we have not found any paper concerning this possibility, this may be just a hypothesis) the case where we use an enzyme bearing a metallic catalyst together with the biological active center. If the problem is in the inactivation of the metal itself and it is not caused by some undesired conformational change of the enzyme structure in the area where the metal is adsorbed, its activity could be recovered. In this case, it might be possible to release the inactivated metal and reload fresh metal batches following a similar protocol to the employed in the preparation of the original artificial organometallic-enzyme complex. If the cause of metal inactivation is a conformational change affecting the area where the metal is assembled and not to the enzyme active center, the solutions will require adding enzyme containing only the metallic active center. (obviously if the accumulation of the substrate of the metal produces some undesired effect as described above).

In this way, although conceptually and academically, the design of enzymes bearing several active centers is of indubitable interest, and their practical implementation should consider how to solve these likely drawbacks, and some other problems that might have been undetected to date.

## 4. Conclusions

The design of enzymes bearing a unique structure and several catalytic activities is of great interest. Starting with natural evolution, similar to fusion enzymes or enzymes bearing a second promiscuous active center, nowadays the tendency is to add catalytic active centers whose activities are quite far from the biological ones, permitting the coupling of biological and metal catalysis to perform the most complex, selective and environmentally friendly cascade reactions. In many instances, as in the development of plurizymes and modified plurizymes, they were only possible by the imagination of the involved researchers and the availability of accurate and powerful enough tools to perform enzyme modelling and design, as well as organic chemistry (to build the suicide inhibitor attached to the organometallic catalysts). These novel tools should be a topic to be strongly developed in the near future, as they may permit the relatively simple coupling of biological and metal catalysis, but to date these tools are clearly under-exploited. This huge potential should not cause researchers to forget some practical problems, such as the necessity of use balanced activities of the involved enzymes to maximize the catalytic potential of the new multiple activity enzymes, or the possibility of very different operational stability of their different elements. While immobilization is usually considered a requisite in most of the industrial applications of enzymes (and most of the enzyme is the result of a sophisticated post-production enzyme chemical modification that can be benefited of the use of solid phase), scarce efforts have been directed to the immobilization of some of these multiple activity enzymes, with the exception of enzymes/metal precipitated and one example of immobilization of an enzyme site directed modified with an organometallic catalyst. It is also likely that this gap may be covered in the upcoming future.

Thus, the application of cascade reactions may be enlarged by the use of biological and metal catalysis but still benefit from the selectivity, reactivity and specificity given by the biological environment. Although academically they are very relevant, the practical advantages of using simultaneously individual natural enzymes and artificial enzyme catalyst bearing just one active center in a cascade reaction versus having both activities in the same unique structure have not been proved in most cases. One exception is the synergy found by the proximity of two organometallic complexes in a modified plurizymes. This synergy between active centers may become an advantage to consider in the design of multi activity enzymes. The future should drive to never stop dreaming of multiple activity enzymes where these advantages may be clearly established, as now we are in the starting of the development of some of the new technologies (e.g., plurizymes).

## Data Availability

Not applicable.

## References

[1] Anastas P., Eghbali N. (2010). Green Chemistry: Principles and Practice. Chem. Soc. Rev..

[2] Sheldon R.A. (2012). Fundamentals of green chemistry: Efficiency in reaction design. Chem. Soc. Rev..

[3] Simon M.O., Li C.J. (2012). Green chemistry oriented organic synthesis in water. Chem. Soc. Rev..

[4] Sheldon R.A. (2008). E factors, green chemistry and catalysis: An odyssey. Chem. Commun..

[5] Sheldon R.A. (2017). The E factor 25 years on: The rise of green chemistry and sustainability. Green Chem..

[6] Sheldon R.A., Arends I.W.C.E., Hanefeld U. (2007). Green Chemistry and Catalysis.

[7] Thompson M.P., Peñafiel I., Cosgrove S.C., Turner N.J. (2019). Biocatalysis using immobilized enzymes in continuous flow for the synthesis of fine chemicals. Org. Process Res. Dev..

[8] Ferreira-Leitão V., Cammarota M., Gonçalves Aguieiras E., Vasconcelos de Sá L., Fernandez-Lafuente R., Freire D. (2017). The protagonism of biocatalysis in green chemistry and its environmental benefits. Catalysts.

[9] Sheldon R.A. (2016). Biocatalysis and green chemistry. Green Biocatalysis.

[10] Zhenming C., Jinhua L., Junhua T. (2007). Biocatalysis for green chemistry and drug development. Prog. Chem..

[11] Schmid A., Dordick J.S., Hauer B., Kiener A., Wubbolts M., Witholt B. (2001). Industrial biocatalysis today and tomorrow. Nature.

[12] Schoemaker H.E., Mink D.L., WubboLts M.G. (2003). Dispelling the myths-Biocatalysis in industrial synthesis. Science.

[13] Sheldon R.A., Woodley J.M. (2018). Role of biocatalysis in sustainable chemistry. Chem. Rev..

[14] Pollard D.J., Woodley J.M. (2007). Biocatalysis for pharmaceutical intermediates: The future is now. Trends Biotechnol..

[15] Reetz M.T. (2013). Biocatalysis in organic chemistry and biotechnology: Past, present, and future. J. Am. Chem. Soc..

[16] Bilal T., Malik B., Hakeem K.R. (2018). Metagenomic analysis of uncultured microorganisms and their enzymatic attributes. J. Microbiol. Methods.

[17] Martínez-Martínez M., Bargiela R., Ferrer M., Brahmachari G., Demain A.L., Adrio J.L. (2017). Metagenomics and the Search for Industrial Enzymes. Biotechnology of Microbial Enzymes: Production, Biocatalysis and Industrial Applications.

[18] Wilson M.C., Piel J. (2013). Metagenomic approaches for exploiting uncultivated bacteria as a resource for novel biosynthetic enzymology. Chem. Biol..

[19] Xing M.N., Zhang X.Z., Huang H. (2012). Application of metagenomic techniques in mining enzymes from microbial communities for biofuel synthesis. Biotechnol. Adv..

[20] Fernández-Arrojo L., Guazzaroni M.-E., López-Cortés N., Beloqui A., Ferrer M. (2010). Metagenomic era for biocatalyst identification. Curr. Opin. Biotechnol..

[21] Lorenz P., Eck J. (2005). Metagenomics and industrial applications. Nat. Rev. Microbiol..

[22] Romero P.A., Arnold F.H. (2009). Exploring protein fitness landscapes by directed evolution. Nat. Rev. Mol. Cell Biol..

[23] Eijsink V.G.H., GÅseidnes S., Borchert T.V., Van Den Burg B. (2005). Directed evolution of enzyme stability. Biomol. Eng..

[24] Arnold F.H. (2009). How proteins adapt: Lessons from directed evolution. Cold Spring Harb. Symp. Quant. Biol..

[25] Xiong W., Liu B., Shen Y., Jing K., Savage T.R. (2021). Protein engineering design from directed evolution to de novo synthesis. Biochem. Eng. J..

[26] Nirantar S.R. (2021). Directed evolution methods for enzyme engineering. Molecules.

[27] Drufva E.E., Hix E.G., Bailey C.B. (2020). Site directed mutagenesis as a precision tool to enable synthetic biology with engineered modular polyketide synthases. Synth. Syst. Biotechnol..

[28] de Graaf C., Oostenbrink C., Keizers P.H.J., van Vugt-Lussenburg B.M.A., van Waterschoot R.A.B., Tschirret-Guth R.A., Commandeur J.N.M., Vermeulen N.P.E. (2007). Molecular modeling-guided site-directed mutagenesis of cytochrome P450 2D6. Curr. Drug Metab..

[29] Boutureira O., Bernardes G.J.L. (2015). Advances in chemical protein modification. Chem. Rev..

[30] Spicer C.D., Davis B.G. (2014). Selective chemical protein modification. Nat. Commun..

[31] Chalker J.M., Bernardes G.J.L., Lin Y.A., Davis B.G. (2009). Chemical modification of proteins at cysteine: Opportunities in chemistry and biology. Chem. Asian J..

[32] Van Kasteren S.I., Kramer H.B., Jensen H.H., Campbell S.J., Kirkpatrick J., Oldham N.J., Anthony D.C., Davis B.G. (2007). Expanding the diversity of chemical protein modification allows post-translational mimicry. Nature.

[33] Mateo C., Palomo J.M., Fernandez-Lorente G., Guisan J.M., Fernandez-Lafuente R. (2007). Improvement of enzyme activity, stability and selectivity via immobilization techniques. Enzym. Microb. Technol..

[34] Sheldon R.A., van Pelt S. (2013). Enzyme immobilisation in biocatalysis: Why, what and how. Chem. Soc. Rev..

[35] Rodrigues R.C., Ortiz C., Berenguer-Murcia Á., Torres R., Fernández-Lafuente R. (2013). Modifying enzyme activity and selectivity by immobilization. Chem. Soc. Rev..

[36] Garcia-Galan C., Berenguer-Murcia Á., Fernandez-Lafuente R., Rodrigues R.C. (2011). Potential of different enzyme immobilization strategies to improve enzyme performance. Adv. Synth. Catal..

[37] Di Cosimo R., Mc Auliffe J., Poulose A.J., Bohlmann G. (2013). Industrial use of immobilized enzymes. Chem. Soc. Rev..

[38] Rodrigues R.C., Berenguer-Murcia Á., Carballares D., Morellon-Sterling R., Fernandez-Lafuente R. (2021). Stabilization of enzymes via immobilization: Multipoint covalent attachment and other stabilization strategies. Biotechnol. Adv..

[39] Schwizer F., Okamoto Y., Heinisch T., Gu Y., Pellizzoni M.M., Lebrun V., Reuter R., Köhler V., Lewis J.C., Ward T.R. (2018). Artificial metalloenzymes: Reaction scope and optimization strategies. Chem. Rev..

[40] Lewis J.C. (2013). Artificial metalloenzymes and metallopeptide catalysts for organic synthesis. ACS Catal..

[41] Steinreiber J., Ward T.R. (2008). Artificial metalloenzymes as selective catalysts in aqueous media. Coord. Chem. Rev..

[42] Rosati F., Roelfes G. (2010). Artificial metalloenzymes. ChemCatChem.

[43] Matsuo T., Miyake T., Hirota S. (2019). Recent developments on creation of artificial metalloenzymes. Tetrahedron Lett..

[44] Thompson Z., Cowan J.A. (2020). Artificial metalloenzymes: Recent developments and innovations in bioinorganic catalysis. Small.

[45] Ren X., Fasan R. (2021). Engineered and artificial metalloenzymes for selective C–H functionalization. Curr. Opin. Green Sustain. Chem..

[46] Large B., Baranska N.G., Booth R.L., Wilson K.S., Duhme-Klair A.-K. (2021). Artificial metalloenzymes: The powerful alliance between protein scaffolds and organometallic catalysts. Curr. Opin. Green Sustain. Chem..

[47] Serafim L.F., Wang L., Rathee P., Yang J., Frenk Knaul H.S., Prabhakar R. (2021). Remediation of environmentally hazardous organophosphates by artificial metalloenzymes. Curr. Opin. Green Sustain. Chem..

[48] Rousselot-Pailley P., Bochot C., Marchi-Delapierre C., Jorge-Robin A., Martin L., Fontecilla-Camps J.C., Cavazza C., Ménage S. (2009). The protein environment drives selectivity for sulfide oxidation by an artificial metalloenzyme. ChemBioChem.

[49] Ortega-Carrasco E., Lledós A., Maréchal J.-D. (2014). Unravelling novel synergies between organometallic and biological partners: A quantum mechanics/molecular mechanics study of an artificial metalloenzyme. J. R. Soc. Interface.

[50] Pordea A., Ward T. (2009). Artificial metalloenzymes: Combining the best features of homogeneous and enzymatic catalysis. Synlett.

[51] Davies C.L., Dux E.L., Duhme-Klair A.-K. (2009). Supramolecular interactions between functional metal complexes and proteins. Dalt. Trans..

[52] Ricca E., Brucher B., Schrittwieser J.H. (2011). Multi-enzymatic cascade reactions: Overview and perspectives. Adv. Synth. Catal..

[53] Guterl J.-K., Garbe D., Carsten J., Steffler F., Sommer B., Reiße S., Philipp A., Haack M., Rühmann B., Koltermann A. (2012). Cell-free metabolic engineering: Production of chemicals by minimized reaction cascades. ChemSusChem.

[54] Sperl J.M., Sieber V. (2018). Multienzyme cascade reactions—Status and recent advances. ACS Catal..

[55] Lopez-Gallego F., Schmidt-Dannert C. (2010). Multi-enzymatic synthesis. Curr. Opin. Chem. Biol..

[56] Velasco-Lozano S., López-Gallego F. (2018). Wiring step-wise reactions with immobilized multi-enzyme systems. Biocatal. Biotransformation.

[57] Arana-Peña S., Carballares D., Berenguer-Murcia Á., Alcántara A.R., Rodrigues R.C., Fernandez-Lafuente R. (2020). One pot use of combilipases for full modification of oils and fats: Multifunctional and heterogeneous substrates. Catalysts.

[58] Arana-Peña S., Carballares D., Morellon-Sterlling R., Berenguer-Murcia Á., Alcántara A.R., Rodrigues R.C., Fernandez-Lafuente R. (2021). Enzyme co-immobilization: Always the biocatalyst designers’ choice…or not?. Biotechnol. Adv..

[59] Hasan F., Shah A.A., Hameed A. (2006). Industrial applications of microbial lipases. Enzym. Microb. Technol..

[60] Jaeger K.-E., Eggert T. (2002). Lipases for biotechnology. Curr. Opin. Biotechnol..

[61] Jaeger K.E., Reetz M.T. (1998). Microbial lipases form versatile tools for biotechnology. Trends Biotechnol..

[62] Aharoni A., Gaidukov L., Khersonsky O., Gould S.M.Q., Roodveldt C., Tawfik D.S. (2005). The “evolvability” of promiscuous protein functions. Nat. Genet..

[63] O’Brien P.J., Herschlag D. (1999). Catalytic promiscuity and the evolution of new enzymatic activities. Chem. Biol..

[64] Khersonsky O., Roodveldt C., Tawfik D.S. (2006). Enzyme promiscuity: Evolutionary and mechanistic aspects. Curr. Opin. Chem. Biol..

[65] Kapoor M., Gupta M.N. (2012). Lipase promiscuity and its biochemical applications. Process Biochem..

[66] Zhang Y., Zhao Y., Gao X., Jiang W., Li Z., Yao Q., Yang F., Wang F., Liu J. (2019). Kinetic model of the enzymatic Michael addition for synthesis of mitomycin analogs catalyzed by immobilized lipase from *T. laibacchii*. Mol. Catal..

[67] Zhao Z., Zhang L., Li F., Tang X., Ma Y., Wang C., Wang Z., Zhao R., Wang L. (2017). A novel oxidation of salicyl alcohols catalyzed by lipase. Catalysts.

[68] Zhang L., Li F., Wang C., Zheng L., Wang Z., Zhao R., Wang L. (2017). Lipase-mediated amidation of anilines with 1,3-diketones via C–C bond cleavage. Catalysts.

[69] Lõpez-Iglesias M., Gotor-Fernández V. (2015). Recent Advances in Biocatalytic Promiscuity: Hydrolase-Catalyzed Reactions for Nonconventional Transformations. Chem. Rec..

[70] Bornscheuer U.T., Kazlauskas R.J. (2004). Catalytic promiscuity in biocatalysis: Using old enzymes to form new bonds and follow new pathways. Angew. Chem. Int. Ed..

[71] Hult K., Berglund P. (2007). Enzyme promiscuity: Mechanism and applications. Trends Biotechnol..

[72] Busto E., Gotor-Fernández V., Gotor V. (2010). Hydrolases: Catalytically promiscuous enzymes for non-conventional reactions in organic synthesis. Chem. Soc. Rev..

[73] Humble M.S., Berglund P. (2011). Biocatalytic promiscuity. Eur. J. Org. Chem..

[74] Izquierdo D.F., Barbosa O., Isabel Burguete M., Lozano P., Luis S.V., Fernandez-Lafuente R., García-Verdugo E. (2014). Tuning lipase B from *Candida antarctica* C-C bond promiscuous activity by immobilization on poly-styrene-divinylbenzene beads. RSC Adv..

[75] Taglieber A., Höbenreich H., Carballeira J.D., Mondière R.J.G., Reetz M.T. (2007). Alternate-site enzyme promiscuity. Angew. Chem. Int. Ed..

[76] Xiang Y., Song J., Zhang Y., Yang D.C., Guan Z., He Y.H. (2016). Enzyme-Catalyzed Asymmetric Domino Thia-Michael/Aldol Condensation Using Pepsin. J. Org. Chem..

[77] Armisén P., Mateo C., Cortés E., Barredo J.L., Salto F., Diez B., Rodés L., García J.L., Fernández-Lafuente R., Guisán J.M. (1999). Selective adsorption of poly-His tagged glutaryl acylase on tailor-made metal chelate supports. J. Chromatogr. A.

[78] Kumar A., Wahlund P.-O., Kepka C., Galaev I.Y., Mattiasson B. (2003). Purification of histidine-tagged single-chain Fv-antibody fragments by metal chelate affinity precipitation using thermoresponsive copolymers. Biotechnol. Bioeng..

[79] Wijekoon C.J.K., Ukuwela A.A., Wedd A.G., Xiao Z. (2016). Evaluation of employing poly-lysine tags versus poly-histidine tags for purification and characterization of recombinant copper-binding proteins. J. Inorg. Biochem..

[80] García J.L., Sánchez-Beato A.R., Medrano F.J., López R. (1998). Versatility of choline-binding domain. Microb. Drug Resist..

[81] Terpe K. (2003). Overview of tag protein fusions: From molecular and biochemical fundamentals to commercial systems. Appl. Microbiol. Biotechnol..

[82] Bolivar J.M., Nidetzky B. (2012). Positively charged mini-protein Z_basic2_ as a highly efficient silica binding module: Opportunities for enzyme immobilization on unmodified silica supports. Langmuir.

[83] Bolivar J.M., Nidetzky B. (2012). Oriented and selective enzyme immobilization on functionalized silica carrier using the cationic binding module Z_basic2_: Design of a heterogeneous D-amino acid oxidase catalyst on porous glass. Biotechnol. Bioeng..

[84] Kweon D.H., Kim S.G., Han N.S., Lee J.H., Chung K.M., Seo J.H. (2005). Immobilization of *Bacillus macerans* cyclodextrin glycosyltransferase fused with poly-lysine using cation exchanger. Enzym. Microb. Technol..

[85] Wiesbauer J., Bolivar J.M., Mueller M., Schiller M., Nidetzky B. (2011). Oriented immobilization of enzymes made fit for applied biocatalysis: Non-covalent attachment to anionic supports using Z_basic2_ module. ChemCatChem.

[86] Nilsson B., Abrahmsén L., Uhlén M. (1985). Immobilization and purification of enzymes with staphylococcal protein A gene fusion vectors. EMBO J..

[87] Richins R.D., Mulchandani A., Chen W. (2000). Expression, immobilization, and enzymatic characterization of cellulose-binding domain-organophosphorus hydrolase fusion enzymes. Biotechnol. Bioeng..

[88] Barbosa O., Ortiz C., Berenguer-Murcia Á., Torres R., Rodrigues R.C., Fernandez-Lafuente R. (2015). Strategies for the one-step immobilization–purification of enzymes as industrial biocatalysts. Biotechnol. Adv..

[89] Madoz J., Kuznetzov B.A., Medrano F.J., Garcia J.L., Fernandez V.M. (1997). Functionalization of gold surfaces for specific and reversible attachment of a fused *β*-galactosidase and choline-receptor protein. J. Am. Chem. Soc..

[90] Sanchez-Puelles J.M., Sanz J.M., Garcia J.L., Garcia E. (1992). Immobilization and single-step purification of fusion proteins using DEAE-cellulose. Eur. J. Biochem..

[91] Qin Z., Lin S., Qiu Y., Chen Q., Zhang Y., Zhou J., Zhao L. (2019). One-step immobilization-purification of enzymes by carbohydrate-binding module family 56 tag fusion. Food Chem..

[92] Moldes C., García J.L., García P. (2004). Construction of a chimeric thermostable pyrophosphatase to facilitate its purification and immobilization by using the choline-binding tag. Appl. Environ. Microbiol..

[93] Munro A.W., Girvan H.M., McLean K.J. (2007). Cytochrome P450–redox partner fusion enzymes. Biochim. Biophys. Acta-Gen. Subj..

[94] Yourno J., Kohno T., Roth J.R. (1970). Enzyme evolution: Generation of a bifunctional enzyme by fusion of adjacent genes. Nature.

[95] Tsoka S., Ouzounis C.A. (2000). Prediction of protein interactions: Metabolic enzymes are frequently involved in gene fusion. Nat. Genet..

[96] Kan S., Aoyagi-Scharber M., Le S.Q., Vincelette J., Ohmi K., Bullens S., Wendt D.J., Christianson T.M., Tiger P.M.N., Brown J.R. (2014). Delivery of an enzyme-IGFII fusion protein to the mouse brain is therapeutic for mucopolysaccharidosis type IIIB. Proc. Natl. Acad. Sci. USA.

[97] Albertsen L., Chen Y., Bach L.S., Rattleff S., Maury J., Brix S., Nielsen J., Mortensen U.H. (2011). Diversion of flux toward sesquiterpene production in *Saccharomyces cerevisiae* by fusion of host and heterologous enzymes. Appl. Environ. Microbiol..

[98] Lee D., Lloyd N.D.R., Pretorius I.S., Borneman A.R. (2016). Heterologous production of raspberry ketone in the wine yeast *Saccharomyces cerevisiae* via pathway engineering and synthetic enzyme fusion. Microb. Cell Fact..

[99] Liu H.-H., Wang C., Lu X.-Y., Huang H., Tian Y., Ji X.-J. (2019). Improved production of arachidonic acid by combined pathway engineering and synthetic enzyme fusion in *Yarrowia lipolytica*. J. Agric. Food Chem..

[100] Rabeharindranto H., Castaño-Cerezo S., Lautier T., Garcia-Alles L.F., Treitz C., Tholey A., Truan G. (2019). Enzyme-fusion strategies for redirecting and improving carotenoid synthesis in *S. cerevisiae*. Metab. Eng. Commun..

[101] Camagna M., Grundmann A., Bär C., Koschmieder J., Beyer P., Welsch R. (2019). Enzyme fusion removes competition for geranylgeranyl diphosphate in carotenogenesis. Plant Physiol..

[102] Nogueira M., Enfissi E.M.A., Welsch R., Beyer P., Zurbriggen M.D., Fraser P.D. (2019). Construction of a fusion enzyme for astaxanthin formation and its characterisation in microbial and plant hosts: A new tool for engineering ketocarotenoids. Metab. Eng..

[103] Harikrishna J.A., Black S.M., Szklarz G.D., Miller W.L. (1993). Construction and function of fusion enzymes of the human cytochrome P450scc system. DNA Cell Biol..

[104] Seo H.S., Koo Y.J., Lim J.Y., Song J.T., Kim C.H., Kim J.K., Lee J.S., Choi Y. (2000). Do Characterization of a bifunctional enzyme fusion of trehalose-6-phosphate synthetase and trehalose-6-phosphate phosphatase of *Escherichia coli*. Appl. Environ. Microbiol..

[105] Bulow L. (1987). Characterization of an artificial bifunctional enzyme, beta-galactosidase/galactokinase, prepared by gene fusion. Eur. J. Biochem..

[106] Roberts I.N., Jeenes D.J., MacKenzie D.A., Wilkinson A.P., Sumner I.G., Archer D.B. (1992). Heterologous gene expression in *Aspergillus niger*: A glucoamylase-porcine pancreatic prophospholipase A2 fusion protein is secreted and processed to yield mature enzyme. Gene.

[107] Du L., Cui X., Li H., Wang Y., Fan L., He R., Jiang F., Yu A., Xiao D., Ma L. (2021). Enhancing the enzymatic hydrolysis efficiency of lignocellulose assisted by artificial fusion enzyme of swollenin-xylanase. Ind. Crops Prod..

[108] Xia Y., Wu Z., He R., Gao Y., Qiu Y., Cheng Q., Ma X., Wang Z. (2021). Simultaneous degradation of two mycotoxins enabled by a fusion enzyme in food-grade recombinant *Kluyveromyces lactis*. Bioresour. Bioprocess..

[109] Liao L., Zhang Y., Wang Y., Fu Y., Zhang A., Qiu R., Yang S., Fang B. (2021). Construction and characterization of a novel glucose dehydrogenase-leucine dehydrogenase fusion enzyme for the biosynthesis of l-tert-leucine. Microb. Cell Fact..

[110] Fabara A.N., Fraaije M.W. (2020). Production of indigo through the use of a dual-function substrate and a bifunctional fusion enzyme. Enzym. Microb. Technol..

[111] Mourelle-Insua Á., Aalbers F.S., Lavandera I., Gotor-Fernández V., Fraaije M.W. (2019). What to sacrifice? Fusions of cofactor regenerating enzymes with Baeyer-Villiger monooxygenases and alcohol dehydrogenases for self-sufficient redox biocatalysis. Tetrahedron.

[112] Baklouti Z., Delattre C., Pierre G., Gardarin C., Abdelkafi S., Michaud P., Dubessay P. (2020). Biochemical characterization of a bifunctional enzyme constructed by the fusion of a glucuronan lyase and a chitinase from *Trichoderma* sp.. Life.

[113] Bülow L., Ljungcrantz P., Mosbach K. (1985). Preparation of a soluble bifunctional enzyme by gene fusion. Nat. Biotechnol..

[114] Lu P., Feng M.-G. (2008). Bifunctional enhancement of a *β*-glucanase-xylanase fusion enzyme by optimization of peptide linkers. Appl. Microbiol. Biotechnol..

[115] Ying X., Wang C., Shao S., Wang Q., Zhou X., Bai Y., Chen L., Lu C., Zhao M., Wang Z. (2020). Efficient oxidation of methyl glycolate to methyl glyoxylate using a fusion enzyme of glycolate oxidase, catalase and hemoglobin. Catalysts.

[116] Elleuche S. (2015). Bringing functions together with fusion enzymes—from nature’s inventions to biotechnological applications. Appl. Microbiol. Biotechnol..

[117] Aalbers F.S., Fraaije M.W. (2019). Enzyme fusions in biocatalysis: Coupling reactions by pairing enzymes. ChemBioChem.

[118] Gudiukaite R., Gricajeva A. (2017). Microbial lipolytic fusion enzymes: Current state and future perspectives. World J. Microbiol. Biotechnol..

[119] Bering L., Thompson J., Micklefield J. (2022). New reaction pathways by integrating chemo- and biocatalysis. Trends Chem..

[120] Palomo J.M. (2021). Artificial enzymes with multiple active sites. Curr. Opin. Green Sustain. Chem..

[121] Rodriguez-Abetxuko A., Muñumer P., Okuda M., Calvo J., Knez M., Beloqui A. (2020). Nanoconfined (bio)catalysts as efficient glucose-responsive nanoreactors. Adv. Funct. Mater..

[122] Mittal A.K., Chisti Y., Banerjee U.C. (2013). Synthesis of metallic nanoparticles using plant extracts. Biotechnol. Adv..

[123] Mashwani Z.-R., Khan T., Khan M.A., Nadhman A. (2015). Synthesis in plants and plant extracts of silver nanoparticles with potent antimicrobial properties: Current status and future prospects. Appl. Microbiol. Biotechnol..

[124] Vetchinkina E.P., Loshchinina E.A., Vodolazov I.R., Kursky V.F., Dykman L.A., Nikitina V.E. (2017). Biosynthesis of nanoparticles of metals and metalloids by basidiomycetes. Preparation of gold nanoparticles by using purified fungal phenol oxidases. Appl. Microbiol. Biotechnol..

[125] Patil M.P., Kim G.-D. (2018). Marine microorganisms for synthesis of metallic nanoparticles and their biomedical applications. Colloids Surf. B Biointerfaces.

[126] Sharma P., Pant S., Rai S., Yadav R.B., Sharma S., Dave V. (2018). Green synthesis and characterization of silver nanoparticles by *Allium cepa* L. to produce silver nano-coated fabric and their antimicrobial evaluation. Appl. Organomet. Chem..

[127] Baghayeri M., Mahdavi B., Hosseinpor-Mohsen Abadi Z., Farhadi S. (2018). Green synthesis of silver nanoparticles using water extract of *Salvia leriifolia*: Antibacterial studies and applications as catalysts in the electrochemical detection of nitrite. Appl. Organomet. Chem..

[128] Wang W., Zhang B., Liu Q., Du P., Liu W., He Z. (2018). Biosynthesis of palladium nanoparticles using *Shewanella loihica* PV-4 for excellent catalytic reduction of chromium(vi). Environ. Sci. Nano.

[129] Zhang X., Yan S., Tyagi R.D., Surampalli R.Y. (2011). Synthesis of nanoparticles by microorganisms and their application in enhancing microbiological reaction rates. Chemosphere.

[130] Boroumand Moghaddam A., Namvar F., Moniri M., Md Tahir P., Azizi S., Mohamad R. (2015). Nanoparticles biosynthesized by fungi and yeast: A review of their preparation, properties, and medical applications. Molecules.

[131] Philip D. (2009). Biosynthesis of Au, Ag and Au–Ag nanoparticles using edible mushroom extract. Spectrochim. Acta Part A Mol. Biomol. Spectrosc..

[132] Willner I., Baron R., Willner B. (2006). Growing metal nanoparticles by enzymes. Adv. Mater..

[133] Palomo J.M. (2019). Nanobiohybrids: A new concept for metal nanoparticles synthesis. Chem. Commun..

[134] Chinnadayyala S.R., Santhosh M., Singh N.K., Goswami P. (2015). Alcohol oxidase protein mediated in-situ synthesized and stabilized gold nanoparticles for developing amperometric alcohol biosensor. Biosens. Bioelectron..

[135] Li X., Fu C., Luo L., Ge J. (2022). Design of enzyme-metal hybrid catalysts for organic synthesis. Cell Rep. Phys. Sci..

[136] Cheng G., Wu Q., Jiang C. (2022). Synthesis of highly active enzyme-metal nanohybrids and uncovering the design rules. Enzym. Microb. Technol..

[137] Liu Y., Liu P., Gao S., Wang Z., Luan P., González-Sabín J., Jiang Y. (2021). Construction of chemoenzymatic cascade reactions for bridging chemocatalysis and biocatalysis: Principles, strategies and prospective. Chem. Eng. J..

[138] Heuson E., Froidevaux R., Itabaiana I., Wojcieszak R., Capron M., Dumeignil F. (2021). Optimisation of catalysts coupling in multi-catalytic hybrid materials: Perspectives for the next revolution in catalysis. Green Chem..

[139] Filice M., Marciello M., Morales M.D.P., Palomo J.M. (2013). Synthesis of heterogeneous enzyme–metal nanoparticle biohybrids in aqueous media and their applications in C–C bond formation and tandem catalysis. Chem. Commun..

[140] Barros H.R., Tanaka L.Y., da Silva R.T.P., Santiago-Arcos J., Torresi S.I.C., López-Gallego F. (2021). Assembly of nano-biocatalyst for the tandem hydrolysis and reduction of p-nitrophenol esters. Part. Part. Syst. Charact..

[141] Naapuri J.M., Åberg G.A., Palomo J.M., Deska J. (2021). Arylative allenol cyclization via sequential one-pot enzyme & palladium catalysis. ChemCatChem.

[142] Carrasco-López C., Godoy C., de las Rivas B., Fernández-Lorente G., Palomo J.M., Guisán J.M., Fernández-Lafuente R., Martínez-Ripoll M., Hermoso J.A. (2009). Activation of bacterial thermo alkalophilic lipases is spurred by dramatic structural rearrangements. J. Biol. Chem..

[143] Filice M., Romero O., Gutiérrez-Fernández J., de las Rivas B., Hermoso J.A., Palomo J.M. (2015). Synthesis of a heterogeneous artificial metallolipase with chimeric catalytic activity. Chem. Commun..

[144] Romero O., de las Rivas B., Lopez-Tejedor D., Palomo J.M. (2018). Effect of site-specific peptide-tag labeling on the biocatalytic properties of thermoalkalophilic lipase from *Geobacillus thermocatenulatus*. ChemBioChem.

[145] Mayer C., Dulson C., Reddem E., Thunnissen A.-M.W.H., Roelfes G. (2019). Directed evolution of a designer enzyme featuring an unnatural catalytic amino acid. Angew. Chem. Int. Ed..

[146] Burke A.J., Lovelock S.L., Frese A., Crawshaw R., Ortmayer M., Dunstan M., Levy C., Green A.P. (2019). Design and evolution of an enzyme with a non-canonical organocatalytic mechanism. Nature.

[147] Drienovská I., Roelfes G. (2020). Expanding the enzyme universe with genetically encoded unnatural amino acids. Nat. Catal..

[148] Zhou Z., Roelfes G. (2020). Synergistic catalysis in an artificial enzyme by simultaneous action of two abiological catalytic sites. Nat. Catal..

[149] Santiago G., Martínez-Martínez M., Alonso S., Bargiela R., Coscolín C., Golyshin P.N., Guallar V., Ferrer M. (2018). Rational engineering of multiple active sites in an ester hydrolase. Biochemistry.

[150] Martínez-Martínez M., Alcaide M., Tchigvintsev A., Reva O., Polaina J., Bargiela R., Guazzaroni M.-E., Chicote Á., Canet A., Valero F. (2013). Biochemical diversity of carboxyl esterases and lipases from Lake Arreo (Spain): A metagenomic approach. Appl. Environ. Microbiol..

[151] Martínez-Martínez M., Coscolín C., Santiago G., Chow J., Stogios P.J., Bargiela R., Gertler C., Navarro-Fernández J., Bollinger A., Thies S. (2018). Determinants and prediction of esterase substrate promiscuity patterns. ACS Chem. Biol..

[152] Allouche A. (2012). Software News and Updates Gabedit—A Graphical User Interface for Computational Chemistry Softwares. J. Comput. Chem..

[153] Hernández-Ortega A., Borrelli K., Ferreira P., Medina M., Martínez A.T., Guallar V. (2011). Substrate diffusion and oxidation in GMC oxidoreductases: An experimental and computational study on fungal aryl-alcohol oxidase. Biochem. J..

[154] Carlson H.A., Smith R.D., Damm-Ganamet K.L., Stuckey J.A., Ahmed A., Convery M.A., Somers D.O., Kranz M., Elkins P.A., Cui G. (2016). CSAR 2014: A benchmark exercise using unpublished data from pharma. J. Chem. Inf. Model..

[155] Santiago G., de Salas F., Lucas M.F., Monza E., Acebes S., Martinez Á.T., Camarero S., Guallar V. (2016). Computer-aided laccase engineering: Toward biological oxidation of arylamines. ACS Catal..

[156] Lecina D., Gilabert J.F., Guallar V. (2017). Adaptive simulations, towards interactive protein-ligand modeling. Sci. Rep..

[157] Alonso S., Santiago G., Cea-Rama I., Fernandez-Lopez L., Coscolín C., Modregger J., Ressmann A.K., Martínez-Martínez M., Marrero H., Bargiela R. (2020). Genetically engineered proteins with two active sites for enhanced biocatalysis and synergistic chemo- and biocatalysis. Nat. Catal..

[158] Fernández-Lafuente R., Rodriguez V., Guisán J.M. (1998). The coimmobilization of d-amino acid oxidase and catalase enables the quantitative transformation of d-amino acids (d-phenylalanine) into α-keto acids (phenylpyruvic acid). Enzym. Microb. Technol..

[159] Li R., Sun J., Fu Y., Du K., Cai M., Ji P., Feng W. (2016). Immobilization of genetically-modified D-amino acid oxidase and catalase on carbon nanotubes to improve the catalytic efficiency. Catalysts.

[160] Upadhya R., Nagajyothi, Bhat S.G. (2000). Stabilization of D-amino acid oxidase and catalase in permeabilized Rhodotorula gracilis cells and its application for the preparation of α- ketoacids. Biotechnol. Bioeng..

[161] Upadhya R., Nagajyothi H., Bhat S.G. (1999). D-Amino acid oxidase and catalase of detergent permeabilized *Rhodotorula gracilis* cells and its potential use for the synthesis of *α*-keto acids. Process Biochem..

[162] Tan Q., Song Q., Zhang Y., Wei D. (2007). Characterization and application of d-amino acid oxidase and catalase within permeabilized *Pichia pastoris* cells in bioconversions. Appl. Biochem. Biotechnol..

[163] Kornecki J.F., Carballares D., Tardioli P.W., Rodrigues R.C., Berenguer-Murcia Á., Alcántara A.R., Fernandez-Lafuente R. (2020). Enzyme production of d-gluconic acid and glucose oxidase: Successful tales of cascade reactions. Catal. Sci. Technol..

[164] Hernandez K., Berenguer-Murcia A., Rodrigues R.C., Fernandez-Lafuente R. (2012). Hydrogen peroxide in biocatalysis. A dangerous liaison. Curr. Org. Chem..

[165] Mateo C., Chmura A., Rustler S., Van Rantwijk F., Stolz A., Sheldon R.A. (2006). Synthesis of enantiomerically pure *(S)*-mandelic acid using an oxynitrilase-nitrilase bienzymatic cascade: A nitrilase surprisingly shows nitrile hydratase activity. Tetrahedron Asymmetry.

[166] Arana-Peña S., Carballares D., Morellon-Sterling R., Rocha-Martin J., Fernandez-Lafuente R. (2022). The combination of covalent and ionic exchange immobilizations enables the coimmobilization on vinyl sulfone activated supports and the reuse of the most stable immobilized enzyme. Int. J. Biol. Macromol..

[167] Carballares D., Rocha-Martin J., Fernandez-Lafuente R. (2022). Chemical amination of immobilized enzymes for enzyme coimmobilization: Reuse of the most stable immobilized and modified enzyme. Int. J. Biol. Macromol..

[168] Carballares D., Rocha-Martin J., Fernandez-Lafuente R. (2022). Coimmobilization of lipases exhibiting three very different stability ranges. Reuse of the active enzymes and selective discarding of the inactivated ones. Int. J. Biol. Macromol..

[169] Carballares D., Rocha-Martin J., Fernandez-Lafuente R. (2022). Preparation of a six-enzyme multilayer combi-biocatalyst: Reuse of the most stable enzymes after inactivation of the least stable one. ACS Sustain. Chem. Eng..

[170] Peirce S., Virgen-Ortíz J.J., Tacias-Pascacio V.G., Rueda N., Bartolome-Cabrero R., Fernandez-Lopez L., Russo M.E., Marzocchella A., Fernandez-Lafuente R. (2016). Development of simple protocols to solve the problems of enzyme coimmobilization. Application to coimmobilize a lipase and a *β*-galactosidase. RSC Adv..

[171] Zaak H., Kornecki J.F., Siar E.-H., Fernandez-Lopez L., Cortés Corberán V., Sassi M., Fernandez-Lafuente R. (2017). Coimmobilization of enzymes in bilayers using PEI as a glue to reuse the most stable enzyme: Preventing PEI release during inactivated enzyme desorption. Process Biochem..

[172] Morellon-Sterling R., Carballares D., Arana-Peña S., Siar E.-H., Braham S.A., Fernandez-Lafuente R. (2021). Advantages of supports activated with divinyl sulfone in enzyme coimmobilization: Possibility of multipoint covalent immobilization of the most stable enzyme and immobilization via ion exchange of the least stable enzyme. ACS Sustain. Chem. Eng..

[173] Arana-Peña S., Carballares D., Cortés Corberan V., Fernandez-Lafuente R. (2020). Multi-combilipases: Co-immobilizing lipases with very different stabilities combining immobilization via interfacial activation and ion exchange. The reuse of the most stable co-immobilized enzymes after inactivation of the least stable ones. Catalysts.

[174] Arana-Peña S., Mendez-Sanchez C., Rios N.S., Ortiz C., Gonçalves L.R.B., Fernandez-Lafuente R. (2019). New applications of glyoxyl-octyl agarose in lipases co-immobilization: Strategies to reuse the most stable lipase. Int. J. Biol. Macromol..

